# Assessing fear of hypoglycemia in a population-based study among parents of children with type 1 diabetes – psychometric properties of the hypoglycemia fear survey – parent version

**DOI:** 10.1186/1472-6823-15-2

**Published:** 2015-01-19

**Authors:** Anne Haugstvedt, Tore Wentzel-Larsen, Morten Aarflot, Berit Rokne, Marit Graue

**Affiliations:** Department of Nursing, Faculty of Health and Social Sciences, Bergen University College, Post Box 7030, N-5020 Bergen, Norway; Centre for Evidence Based Practice, Bergen University College, Bergen, Norway; Centre for Child and Adolescent Mental Health for Eastern and Southern Norway, Oslo, Norway; Norwegian Centre for Violence and Traumatic Stress Studies, Oslo, Norway; Centre for Clinical Research, Haukeland University Hospital, Bergen, Norway; Department of Research and Development, Haukeland University Hospital, Bergen, Norway; Department of Pediatrics, Haukeland University Hospital, Bergen, Norway; Department of Global Public Health and Primary Care, University of Bergen, Bergen, Norway

**Keywords:** Hypoglycemia, Fear, Parents, HFS, HFS-P, Reliability, Validity

## Abstract

**Background:**

In the treatment of childhood type 1 diabetes, being aware of the parents’ fear of hypoglycemia is important, since the parents’ fear may influence the management of treatment and the children’s blood glucose regulation. The availability of proper instruments to assess the parents’ fear of hypoglycemia is essential. Thus, the aim of this study was to examine the psychometric properties of the Hypoglycemia Fear Survey – Parent version (HFS-P).

**Methods:**

In a Norwegian population-based sample, 176 parents representing 102 children with type 1 diabetes (6–15 years old) completed the HFS-P, comprising a 15-item worry subscale and a 10-item behavior subscale. We performed exploratory and confirmatory factor analysis and further analysis of the scales’ construct validity, content validity and reliability.

**Results:**

The Norwegian version of the HFS-P had an acceptable factor structure and internal consistency for the worry subscale, whereas the structure and internal consistency of the behavior subscale was more questionable. The HFS-P subscales were significantly correlated (from moderately to weakly) with symptoms of emotional distress, as measured by the Hopkins Symptom Checklist – 25 items. The mothers scored higher than fathers on both HFS-P subscales, but the difference was not statistically significant for the worry subscale.

**Conclusions:**

The HFS-P worry subscale seems to be a valid scale for measuring anxiety-provoking aspects of hypoglycemia, and the validity of the HFS-P behavior subscale needs to be investigated further.

## Background

Hypoglycemia is one of the most common acute complications in insulin-treated diabetes [1] and is indicated to be an important limiting factor for glycemic control in type 1 diabetes [2, 3]. Both nocturnal hypoglycemia and hypoglycemia that causes unpleasant symptoms and situations may frighten not only the person with diabetes but also the relatives and parents. In the treatment of children with type 1 diabetes, being aware of the parents’ fear of hypoglycemia is important, since the parents’ fear may influence the management of the treatment of the child. The most common instrument for assessing the fear of hypoglycemia among adults with diabetes is the Hypoglycemia Fear Survey (HFS) [4, 5]. The HFS – Parent version (HFS-P) is the most common instrument to assess fear of hypoglycemia among parents of children with type 1 diabetes. The HFS-P is adapted from the original adult version and comprises worry and behavior subscales [6, 7].

Compared with the adult version of HFS, there is less research on the psychometric properties of the HFS-P. Some previous publications [6–9] have reported adequate reliability for the HFS-P, and one recent publication has reported on the construct validity of the scale [10]. Some studies [6, 9, 11] have identified an association between parental fear of hypoglycemia and poor glycemic control among children with type 1 diabetes. Patton et al. [11] used a modified version of the HFS-P for parents of young children (infants, toddlers and preschool). An association between parental fear of hypoglycemia and poor glycemic control may indicate that the parents’ fear causes long-term negative health effects among children with type 1 diabetes. Thus, valid and reliable instruments are needed to reveal and assess parental fear of hypoglycemia. This study therefore aimed to examine the psychometric properties of the HFS-P, including its factor structure, in a population-based study among both the mothers and the fathers of children with type 1 diabetes in Norway.

## Methods

### Design and participants

In a population-based cross-sectional study we, initially, collected data on the HFS-P from 200 parents representing 115 children 15 years old or younger with type 1 diabetes in one county in Norway [9]. While some of the items in the HFS-P might not be appropriate for the parents of the youngest children (such as items referring to the child being alone or concerns about social embarrassment) [8], the present validation study did not include parents of children younger than 6 years. In total, 176 parents (91 mothers and 85 fathers) of 102 children aged 6–15 years participated in this study. The 102 children constituted 69% of the children 6–15 years old with type 1 diabetes in a specific county in Norway. The 43 children of the nonresponding parents did not differ significantly in mean HbA_1c_ from the children of parents who did respond; 8.4% (SD 1.2) versus 8.2% (SD 1.1) (NGSP/DCCT units) (68.3 mmol/mol (SD 9.8) versus 66.1 (SD 8.9) mmol/mol (SI (IFCC)). Table 1 shows the characteristics of the 102 children included in the study, and Table 2 shows the characteristics of the parents. We collected the data about the child’s age, duration of diabetes, HbA_1c_ value and insulin treatment regimen from medical records. The parents provided information on routines for blood glucose measurements and the frequency of what the parents experienced as problematic hypoglycemic events in the past year. These data showed close to 100% agreement between the reports from a child’s mother and father [9], which confirmed that the sample has a cluster structure.

**Table 1:** **Characteristics of 102 children (aged 6–15 years) with type 1 diabetes**

	***n***(%)	Mean (range)	SD
Boys	52 (51)		
Mean age (years)		11.4 (6.1–15.9)	2.9
Age groups			
6–11 years	57 (56)		
12–15 years	45 (44)		
Age at onset (years)		7.2 (1.1–14.3)	3.3
Diabetes duration (years)		3.9 (0.3–14.2)	2.9
HbA_1c_ (%) (NGSP/DCCT)^*^		8.2 (6.1–11.7)	1.0
Insulin treatment			
Insulin pump	45 (44)		
≥3 injections per day	57 (56)		
Blood glucose measurements^*^ (*n* = 100)			
≤3 times/day	13 (13)		
4–6 times/day	57 (57)		
≥7 times/day	30 (30)		
Monitoring at night^†^ (*n* = 101)			
Every week or more often	24 (24)		
Experienced hypoglycemia^†^ (*n* = 99)			
>7 problematic episodes in the past 12 months	22 (22)		
With unconsciousness, ever (*n* = 101)	24 (24)		
During night, ever (*n* = 100)	71 (71)		

*SI (IFCC): 66.1 (43.2–104.4) mmol/mol.

^†^Mothers’ reports if available; if not, fathers’ reports (data showed close to 100% agreement between mothers’ and fathers’ reports on these items).

**Table 2:** **Characteristics of the 176 participating parents of children with type 1 diabetes**

	Mothers	Fathers
Participants	91 (52)	85 (48)
Mean age (year (SD))	40.2 (5.7)	43.4 (6.3)
Reported to be married or cohabiting (*n* (%))^*^	77 (88)	76 (91)
Reported education at university or university college level (*n* (%))	30 (33)	30 (36)
Full-time employment status (*n* (valid %)^†^	36 (37)	88 (92)

*Valid percentage is used because of considerable missing data. Seven mothers and one father did not report on this variable

^†^Five mothers and one father did not report on this variable.

### Ethical considerations

The information sheets and study questionnaires were distributed by post. Completing and returning the questionnaire was considered as informed consent. The Western Norway Regional Committee for Medical and Health Research Ethics approved the study, which was performed according to the Declaration of Helsinki.

### Questionnaires

The HFS-P used in the study consists of a 15-item worry subscale and a 10-item behavior subscale (Table 3) in accordance with the scale presented by Clarke et al. [6]. The worry subscale items measure anxiety-provoking aspects of hypoglycemia (such as “child not recognizing that he/she is having a reaction” and “child having a reaction while asleep”), and the behavior subscale items measure specific behavior carried out to avoid hypoglycemia (such as “have my child eat large snacks at bedtime” and “allow my child’s blood sugar to be a little high to be on the safe side”). The items are rated on a five-point Likert scale ranging from 1 (never) to 5 (always). The HFS-P subscale scores and total score are obtained by summing the items for the worry subscale (range 15–75), the behavior subscale (range 10–50) and the HFS-P total (range 25–125). Higher scores indicate higher fear of hypoglycemia. Both the total score and subscale scores have previously been recommended for analysis [6, 12, 13]. The HFS-P was translated into Norwegian for this study using the academic translation procedure recommended by WHO (http://www.who.int/substance_abuse/research_tools/translation/en) [9], and we met with experts in the field to agree about the final Norwegian version. Few HFS-P data were missing in this study. Item 1 and 2 in the behavior subscale had missing data from 9 and 8 mothers, respectively. All other items had fewer missing data, mostly between 0 and 4 among both mothers and fathers. All respondents had at least 19 of 25 valid answers. As recommended [14], we based scale scores on the within-person means of the answered items if at least half the items were answered. The results section presents the psychometric properties of the scale.

**Table 3:** **Forced two-factor exploratory factor analysis for the Hypoglycemia Fear Survey –Parent version (HFS-P) worry and behavior subscales**

	Fathers		Mothers	
Worry subscale	Factor 1	Factor 2	Factor 1	Factor 2
1. Child not recognizing/realizing that he/she is having a reaction.	*0.459*	0.318	*0.520*	*0.444*
2. Child not having food, fruit, or juice with him/her.	0.327	0.332	*0.427*	0.196
3. Child feeling dizzy or passing out in public.	*0.565*	0.189	*0.710*	0.141
4. Child having a reaction while asleep.	*0.649*	0.206	*0.725*	-0.106
5. Child embarrassing self or friends/family in a social situation.	*0.461*	-0.060	0.072	*0.572*
6. Child having a reaction while alone.	*0.773*	0.202	*0.733*	0.184
7. Child appearing to be “stupid” or clumsy.	*0.486*	-0.094	0.106	*0.961*
8. Child losing control.	*0.656*	0.026	*0.740*	0.207
9. No one being around to help child during a reaction.	*0.681*	0.148	*0.727*	0.204
10. Child making a mistake or having an accident at school.	*0.512*	0.018	0.317	*0.539*
11. Child getting a bad evaluation at school because of something that happens when his/her sugar is low.	*0.480*	-0.130	0.216	*0.734*
12. Child having seizures or convulsions.	*0.665*	0.326	*0.688*	0.203
13. Child developing long term complications from frequent low blood sugar.	*0.691*	0.228	*0.492*	0.167
14. Child feeling light-headed or faint.	*0.755*	-0.046	*0.579*	0.151
15. Child having an insulin reaction.	*0.615*	0.305	*0.698*	0.025
**Behavior subscale**				
1. Have my child eat large snacks at bedtime.	0.155	0.371	*0.570*	-0.013
2. Avoid having my child being alone when his/her sugar is likely to be low.	0.104	0.178	0.357	0.176
3. Allow my child’s blood sugar to be a little high to be on the safe side.	0.009	*0.693*	*0.561*	0.164
4. Keep my child’s sugar higher when he/she will be alone for a while.	-0.056	*0.751*	*0.514*	0.065
5. Have my child eat something as soon as he/she feels the first sign of low blood sugar.	0.007	*0.584*	0.276	0.089
6. Reduce my child’s insulin when I think his/her sugar is too low.	-0.157	*0.408*	0.238	-0.180
7. Keep my child’s blood sugar higher when he/she plans to be away from me for a while.	0.177	*0.660*	*0.574*	0.102
8. Have my child carry fast-sugar.	-0.087	-0.021	0.064	-0.135
9. Have my child avoid a lot of exercise when I think his/her sugar is low.	0.048	*0.561*	0.099	0.177
10. Check my child’s sugar often when he/she plans to go on an outing.	0.142	0.386	*0.407*	-0.133

The Hopkins Symptom Checklist – 25 items (HSCL-25) was used to investigate convergent validity. HSCL-25 is a general instrument measuring symptoms of anxiety and depression. The HSCL-25 comprises two subscales asking questions about the presence and intensity of anxiety and depression symptoms, respectively (such as headaches, restlessness, dizziness, sleep deprivation, poor appetite and anxiety) during the previous 2 weeks. The HSCL-25 has been shown to be a valid instrument for screening emotional distress among non-psychiatric patient populations [15–17]. For the HSCL-25, scale scores were also based on the within-person means of the answered items if at least half the items were answered. In this study, Cronbach’s alpha for the total scale was 0.88 among fathers and 0.92 among mothers, 0.70 and 0.81 for the anxiety subscale and 0.82 and 0.91 for the depression subscale, respectively.

### Analysis

The expert group participating in the translation consensus meeting explored the content validity of the Norwegian HFS-P. In addition, we conducted pilot testing among 8 parents of children with type 1 diabetes to explore the face validity of the scale. To explore the probability that both HFS-P subscales are dealing with a common superior factor such as fear, we computed Pearson correlations (*r*) between the HFS-P worry subscale scores and the HFS-P behavior subscale scores in mothers and fathers, respectively. We measured the internal consistency reliability of the HFS-P subscales by using Cronbach’s alpha. Values ≥0.70 are regarded as satisfactory [14].

To investigate the construct validity [14] of the HFS-P, we performed first exploratory factor analysis for the total scale, with principal axis factoring and varimax rotation. We used the eigenvalue ≥1 criterion. Further, we tested the scales’ two-factor structure with a beforehand-fixed two-factor solution. We determined the allocation of items to the factors by rotated factor loadings ≥0.4 in absolute value. Finally, we also performed maximum likelihood confirmatory factor analysis to confirm the two-factor solution with one worry subscale and one behavior subscale described by Clarke et al. [6]. All items in the worry and behavior subscales were included in the confirmatory factor analysis. The model fit criteria were root mean square error of approximation (RMSEA; preferably less than 0.08), comparative fit index (CFI; preferably at least 0.95) and Tucker-Lewis index (TLI; preferably at least 0.95). In case of moderately inferior fit, we conducted exploratory post hoc investigation in complete cases.

We checked the HFS-P subscale data for possible floor and/or ceiling effects and deviation from a normal distribution. To investigate convergent and discriminant validity for the HFS-P scales [14], we computed Pearson correlations between HFS-P subscale scores and the HSCL-25 subscale scores and total scale scores measuring symptoms of anxiety and depression (respectively) and general emotional distress. If the HFS-P subscales assess worries and inappropriate behavior related to fear of hypoglycemia, it is reasonable to expect moderate positive correlation between the HFS-P subscale scores and the HSCL-25 scores. To test the HFS-P’s ability to discriminate between groups, we hypothesized HFS-P subscale scores to be higher among mothers than among fathers. We used paired-sample *t*-tests for these analyses.

Finally, to provide additional validity data for the use of the HFS-P, we computed Pearson correlations between the parents’ HFS-P subscale scores and the children’s age, duration of diabetes and HbA_1c_, we performed independent-sample *t*-tests to analyze differences in HFS-P subscale scores between the parents of children using an insulin pump and the parents of children using multiple daily injections, and we performed analysis of variance (ANOVA) to analyze differences in mean HFS-P subscale scores between groups reporting various frequency of problematic hypoglycemic episodes the past year (including the groups “none”, “1–2”, “3–6” and “≥7”) and between groups reporting different daily frequency of blood glucose measurements (including the groups “≤3”, “4–6” and “≥7” measurements per day).

For 74 of the 102 children in this study, both the mother and father answered the study questionnaire. By definition, this must be interpreted as data with a cluster structure. Thus, we considered it most appropriate to perform all analyses, except pairwise comparisons, separately for the mothers’ and the fathers’ reports. We defined statistical significance as *P* < 0.050 and used SPSS versions 21 and 22 and AMOS (IBM SPSS, Armonk, NY, USA) for the analysis.

## Results

### Content and face validity

The piloting of the HFS-P among 8 parents gave no remarks to the content and the wording of the items in the scale. The participants experienced the items as being relevant and appropriate. However, participants in the consensus meeting among translators and experts questioned whether some items within the HFS-P behavior subscale measures both inappropriate behavior related to fear and appropriate behavior to avoid hypoglycemia such as “reduce my child’s insulin when I think his/her sugar is too low” or “have my child eat something as soon as he/she feels the first sign of low blood sugar”. Pearson’s correlations between the parents’ reports on the worry and the behavior subscales indicated relatively weak correlations between the subscales among both mothers (*r* = 0.36, *P* < 0.001) and fathers (*r* = 0.27, *P* < 0.001).

### Reliability

The Norwegian version of the HFS-P showed good internal consistency for the worry subscale, with Cronbach’s alpha being 0.89 among both mothers and fathers. The behavior subscale had somewhat weaker Cronbach’s alpha values, with 0.72 among both mothers and fathers.

### Construct validity

For fathers’ reports, the exploratory factor analysis indicated a possible Haywood case [18], and we therefore excluded the two items with lowest communalities (items 1 and 8) in the behavior subscale from the analysis among the fathers’ reports. All items were included in the analysis among the mothers’ reports. Among the fathers’ worry scores, 14 of 15 items loaded within two factors, with the first factor comprising 10 items reflecting “worries about not being able to help the child or the child being out of control” (items 1, 3, 4, 6, 8, 9 and 12–15) and the second factor comprising 4 items reflecting “worries that the child should suffer from socially embarrassing or unpleasant consequences due to hypoglycemia” (items 5, 7, 10 and 11). Nearly the same results were seen among the mothers’ reports, with 8 items loading for the first factor (items 3, 4, 6, 8, 9, 12, 13 and 15) and 5 items loading for the second factor (items 1, 5, 7, 10 and 11). For the behavior subscale, the analysis among the fathers’ reports showed that 5 of the 8 items included loaded for one factor reflecting “concerns with keeping the child’s blood glucose higher, especially when the parent is going to be away” (item 3, 4, 6, 7, 9) and 2 items loaded for a second factor reflecting “behaviors to prevent and/or treat hypoglycemia” (item 2 and 5). Among the mothers’ behavior scores 4 items loaded for the first factor (items 3, 4, 7 and 10) and 3 items loaded for the second factor (items 2, 5 and 6).

The exploratory factor analysis with two beforehand-fixed factors (as indicated from the authors of the HFS-P) only partly supports the two-factor solution. Concerning the worry subscale, 14 of 15 items among the fathers and 11 of 15 items among the mothers loaded (>0.4) in factor 1 (Table 3). For the behavior subscale, the results were more ambiguous. Six of 10 items among the fathers and 0 of 10 items among the mothers loaded (>0.4) in factor 2.

The confirmatory factor analysis among the fathers’ answers with no modification gave CFI = 0.70, TLI = 0.65 and RMSEA = 0.09. Based on modification indices, we included three error-term covariances in the worry subscale between items 5 and 7, items 5 and 11 and items 7 and 11, and one error-term covariance between items 5 and 8 in the behavior subscale. The fit indices after modification were CFI = 0.77, TLI = 0.81 and RMSEA = 0.07. The confirmatory factor analysis among the mothers’ answers with no modification gave CFI = 0.73, TLI = 0.68 and RMSEA = 0.07. Based on modification indices, we included three error-term covariances in the worry subscale between items 5 and 7, items 7 and 11 and items 12 and 13. The fit indices after modification were CFI = 0.79, TLI = 0.76 and RMSEA = 0.07.

Among the mothers, the HFS-P worry subscale scores were statistically significantly correlated with HSCL-25 total scale scores (*r* = 0.40, *P* < 0.001), HSCL-25 anxiety subscale scores (*r* = 0.42, *P* < 0.001) and depression subscale scores (*r* = 0.35, *P* = 0.001). The correlations between the mothers’ HFS-P behavior subscale scores and HSCL-25 scores were not significant. Among the fathers, the HFS-P worry subscale scores were statistically significantly but more weakly correlated with HSCL-25 total scale scores (*r* = 0.27, *P =* 0.014) and the depression subscale scores (*r* = 0.24, *P* = 0.031). The fathers’ behavior subscale scores were also statistically significantly but weakly correlated with the HSCL-25 total scale scores (*r* = 0.25, *P =* 0.022) and the depression subscale scores (*r* = 0.26, *P* = 0.018). The fathers’ HSCL-25 anxiety subscale scores were not significantly correlated with the HFS-P subscale scores. The paired-sample *t*-tests indicated nonsignificant differences (*P* = 0.321) between mothers’ and fathers’ reports on the worry subscale (37.9 (SD 8.7) versus 36.7 (SD 8.7)) (range 15–75). On the behavior subscale, however, the parents differed statistically significantly (*P* < 0.001). The mothers scored a mean 33.2 (SD 5.9) and the fathers a mean 30.5 (SD 6.0) (range 10–50). No floor or ceiling effects and no substantial skewness were identified for the HFS-P data.

The children’s age was significantly negative correlated with the HFS-P behavior scores among both mothers (*r* = -0.40, *P* < 0.001) and fathers (*r* = -0.24, *P* = 0.028) reports. Behavior scores were also significantly lower among the mothers of children using an insulin pump than among the mothers of children with multiple daily injections (31.3 versus 34.4, *P* = 0.024). We found no statistically significant correlation between the parents’ worry or behavior scores and the children’s duration of diabetes or HbA_1c_ level. Compared with the mothers reporting no problematic hypoglycemic episodes in the past year, the mothers of children reporting 7 or more episodes scored higher on both the worry subscale (42.1 (SD 9.3) versus 36.7 (SD 10.3)) and behavior subscale (34.3 (SD 6.5) versus 31.0 (SD 5.8)). The differences between the groups included were, however, not statistically significant. The same picture was seen among the fathers. Further, the mothers who reported 7 or more blood glucose measurements per day had higher behavior score (36.6 (SD 4.8)) than those reporting 4–6 measurements (32.1 (SD 5.6)) or 3 or less measurements (31.2 (SD 7.5)) per day (*P* = 0.003).

## Discussion

The exploratory factor analysis with two factors fixed in advance indicated an acceptable factor structure for the HFS-P worry subscale, whereas the structure within the HFS-P behavior subscale was more questionable. The analysis indicated, however, the best fit for a two-factor solution for both subscales. The confirmatory factor analysis indicated a need for further improvement and development of the scale. The expected relationship between HFS-P and HSCL-25 and the differences between mothers and fathers indicated adequate convergent and discriminant validity. Internal consistency was satisfactory for the HFS-P worry subscale but somewhat weak for the HFS-P behavior subscale.

In accordance with our results, previous research has also shown somewhat questionable validity for the HFS-P behavior subscale, and authors have questioned whether the HFS-P behavior subscale measures both inappropriate behavior related to fear and appropriate behavior to avoid hypoglycemia [6, 7]. The lower HFS-P behavior score identified among the mothers of children using an insulin pump than among the mothers of children with multiple daily injections and the association between the parents’ behavior scores and the children’s age may indicate appropriate behavior. Using an insulin pump may cause a more predictable blood glucose concentration and less need for preventive behavior to avoid hypoglycemia than using insulin injections does [19]. More preventive behavior to avoid hypoglycemia among the youngest children may be appropriate as well.

The question about measuring both inappropriate and appropriate behavior has also been addressed for the HFS behavior subscale in versions for adults with diabetes [4, 5, 20, 21]. The developers of the scales have recently recommended a three-factor solution for the adult version of HFS [5]. The behavior subscale of the adult version is divided into two subscales in the new recommendation: “maintain high blood glucose” subscale and “avoidance” subscale. This division of the behavior subscale is in accordance with both our findings and the findings and recommendations of Shepard et al. [10]. Both our study and the study of Shepard et al. [10] indicated, in addition, a division of the worry subscale into one factor concerning “worries about not being able to help” and a second factor concerning “worries that the child should suffer from socially embarrassing or unpleasant consequences”.

It is reasonable to question whether the discussion related to the behavior subscale may be related to an unclear and varying definition and understanding of the concept of fear. The diversity between mothers and fathers in the results obtained in the exploratory factor analysis for the behavior subscale, may indicate diversity in understanding of the items between the sexes. In a previous study [22], we found a closer relationship between diabetes-related burden and symptoms of emotional distress among the mothers than among the fathers. Such a difference may cause a different understanding or interpretation of the items in the behavior subscale. The causes for the behavior reported may also differ. An important question is, however, whether the measure of fear only should be related to inappropriate emotional distress and inappropriate behavior, or whether appropriate preventive behavior could be a consequence of appropriate fear. For example, “avoiding having a child be alone when his or her blood sugar is likely to be low” (item 2) may be interpreted as an appropriate behavior related to appropriate fear of more severe hypoglycemia.

The question of appropriateness related to some of the questions in the HFS-P behavior subscale may partly be related to the child’s age. Presuming that a child’s age influences the parents’ answers on some of the items in the behavior subscale is reasonable, although we did not find a statistically significant correlation between age and behavior scale scores. By using the same instrument (but named PFSH), Gonder-Frederick et al*.* [8] identified significantly higher behavior scores among the parents of children 6–8 and 9–11 years old than among the parents of children 12–18 years old, and they concluded that this may be a result of an age-appropriate division of responsibility more than a result of different levels of fear.

The results of the factor analysis performed in this study and the relatively weak correlations identified between the HFS-P worry and behavior subscales among both mothers and fathers may question whether the two subscales really measure a common underlying concept of fear. The authors of the HFS-P have previously recommended using the subscales separately as well as analyzing the total scale [6, 7]. The most important suggestion from the authors, however, is not to exclude the behavior subscale from future research on fear of hypoglycemia, as has been done in some studies because of the questionable validity of the behavior subscale [21]. The behavior subscale may capture important aspects related to blood glucose regulation and both appropriate and inappropriate behavior to prevent hypoglycemia. Our results and the mentioned questions related to the behavior subscale may, however, raise a suggestion for separate analysis of the worry and behavior subscales.

The significantly higher HFS-P behavior score among the mothers of children measuring blood glucose ≥7 times per day compared to those measuring 4–6 or ≤3 times per day may indicate either positive maternal engagement in diabetes management or negative preoccupation and distress. The correlations between worry subscale scores and HSCL-25 total scale scores and anxiety and depression subscale scores identified among the mothers in this study, indicate, however, that the fear measured by the worry subscale is related to symptoms of emotional distress. From our study, we cannot draw conclusions about causal direction between the fear of hypoglycemia and the general symptoms of emotional distress, but the correlation indicates an adequate convergent validity for the HFS-P worry subscale. The differences identified in HFS-P subscale scores between mothers and fathers support adequate discriminant validity of the HFS-P. One could postulate that the differences identified between the parents’ reports could be related to differences in responsibility for management tasks between the parents. In our study, however, between 50% and 70% of the parents reported shared responsibility for various diabetes management tasks.

The Cronbach’s alpha values for the HFS-P in this study indicated acceptable internal consistence reliability for the worry subscale. The results are comparable with previous reports of Gonder-Frederick et al*.* [6–8]. Our study, similar to the more recent study of Gonder-Frederick et al*.* [8] among parents of children of all ages between 6 and 18 years, identified somewhat weaker Cronbach’s alpha values (0.58–0.72) for the behavior subscale, which supports the question of whether the items in the behavior subscale actually measure a common underlying concept.

This study has weaknesses. The sample size was limited, and this may be the reason for the statistically nonsignificant associations identified between the HFS-P subscale scores and more problematic hypoglycemic episodes. In a previous publication using statistics for clustered data, we found a statistically significant association between higher worry subscale scores and a higher frequency of hypoglycemic episodes [9]. Although the study used a population-based sample in one county in western Norway, the sample might not be representative of all parents of children with type 1 diabetes in other cultures and settings of care. Test-retest analysis was not performed. The questions mentioned related to the behavior subscale and the results from the factor analysis indicate a need for further research related to the scale. International collaboration between researchers is recommended for further developing the scale and developing a manual for interpreting results.

## Conclusions

The results of this validation of the Norwegian version of the HFS-P support continued use of the instrument. However, some notification should be made for future research. The HFS-P worry subscale seems to be a valid scale for measuring the anxiety-provoking aspects of hypoglycemia, whereas the validity of the behavior subscale is more questionable. Both the worry and behavior subscales are suggested for future research, but the scales might best be reported separately. A two-factor solution for both subscales should be further investigated, and a manual for interpreting the HFS-P results is required.

## Abbreviations

HFS: Hypoglycemia fear survey
HFS-P: Hypoglycemia fear survey – parent version
HSCL-25: Hopkins symptom checklist – 25 items
HbA_1c_: Glycated hemoglobin.
